# Pooled Analysis of Complications with Transvenous ICD Compared to Subcutaneous ICD in Patients with Catecholaminergic Polymorphic Ventricular Arrhythmia

**DOI:** 10.3390/jpm12040536

**Published:** 2022-03-28

**Authors:** Henrik Eckert, Ibrahim El-Battrawy, Michael Veith, Gretje Roterberg, Jacqueline Kowitz, Siegfried Lang, Xiaobo Zhou, Ibrahim Akin, Andreas Mügge, Assem Aweimer

**Affiliations:** 1 First Department of Medicine, Faculty of Medicine, University Medical Centre Mannheim (UMM), University of Heidelberg, 68167 Mannheim, Germany; henrik.eckert@gmx.de (H.E.); michael.veith1@gmx.de (M.V.); gretje.roterberg@web.de (G.R.); jm.k@live.com (J.K.); siegfried.lang@umm.de (S.L.); xiaobo.zhou@medma.uni-heidelberg.de (X.Z.); ibrahim.akin@umm.de (I.A.); 2 DZHK (German Center for Cardiovascular Research), Partner Site, Heidelberg-Mannheim, 68167 Mannheim, Germany; 3 Bergmannsheil Bochum, Medical Clinic II, Department of Cardiology and Angiology, Ruhr University, 44789 Bochum, Germany; andreas.muege@bergmannsheil.de (A.M.); assem.aweimer@bergmannsheil.de (A.A.)

**Keywords:** catecholaminergic polymorphic ventricular tachycardia, sudden cardiac death, ICD-complications, subcutaneous defibrillator

## Abstract

Background: Catecholaminergic polymorphic ventricular tachycardia (CPVT) is associated with arrhythmic events which may lead to sudden cardiac death (SCD). A leading therapy for CPVT besides medical treatment with beta-blockers is the use of an implantable cardioverter-defibrillator (ICD). For this paper we compared data from a pooled analysis to get further evidence about the complications of transvenous and subcutaneous ICDs. Methods: We gathered data from a search of PubMed, Web of Science, Cochrane Library and Cinahl. For our analysis, we chose 30 studies with a total number of 784 patients. We compared the data regarding complications caused by different ICD device types. Results: During a mean follow up of 38.9 months for the patients with ICD implantation (*n* = 337), data showed a complication rate of 101 (30%). A total of 330 (98%) of them received a transvenous-ICD (T-ICD) and 7 (2%) a subcutaneous-ICD (S-ICD). A total of 97 (29.4%) of the T-ICD patients and 4 (57.1%) of the S-ICD patients had at least one complication. Of the 234 complications that occurred in T-ICD patients 152 (65%) were inappropriate shocks due to supraventricular arrhythmias, T/R-wave oversensing or electrode defect, 26 (11.1%) lead fracture/failure, 1 (0.4%) electrode defect, 46 were (19.7%) events of electrical storms, 1 (0.4%) thromboembolic event, 2 (0.8%) cases of endocarditis and 6 (2.6%) infections of the ICD-pocket. Ten (100%) of the complications for the four patients with the S-ICD were an event of an inappropriate shock due to supraventricular arrhythmias, T/R-wave oversensing or electrode defect. Conclusion: Subcutaneous ICDs (S-ICD) show a certain advantage over T-ICDs regarding lead-related complications. Nevertheless, they still show problems with inappropriate shocks and other ICD related complications. Therefore, a case-by-case decision is advised, but the continuous improvement of S-ICD might make it an overall advantageous therapy option in the future.

## 1. Introduction

Catecholaminergic polymorphic ventricular tachycardia (CPVT) is an inherited arrhythmogenic disorder characterized by ventricular tachyarrhythmias (VAs) in a structurally normal heart. This rare channelopathy manifests especially in childhood and adolescence with symptoms such as syncope and/or sudden cardiac death (SCD). The risk of cardiac events is high, and the incidence of SCD has been estimated to be around 6.4% to 13% over an eight-year period [1,2].

Besides anti-arrhythmic drugs such as beta blockers, which reduce the risk of VAs, and Flecainide, which reduces the occurrence of VAs further, a leading therapy for patients with CPVT is the use of an implantable cardioverter-defibrillator (ICD) [3].

Transvenous ICD systems (T-ICD) are most used in the therapy of hereditary channelopathies, but subcutaneous ICDs (S-ICD) have become an emerging alternative in the last years. Although T-ICD systems are proven to be effective in the prevention of SCD, they are still associated with a risk for long-term complications such as endocarditis, lead dislodgement and lead failure, and consequently with considerable lethality. In particular, the T-ICD leads show an annual failure rate of up to 20% for 8-year-old systems [4]. The S-ICD (Boston Scientific, Natick, MA, USA) remains outside of the cardiac space and vasculature and therefore shows a different spectrum of complications. Recently published data in small cohorts of patients with channelopathies show promising results of S-ICDs as compared to T-ICDs [5].

## 2. Methods

In this analysis, we aimed to gather a large number of patients who were diagnosed with CPVT and received treatment with an ICD device. Thus, we looked for studies that provided us with detailed information about the treatment and complications under ICD therapy followed up for a considerable amount of time from a clinical perspective.

### Pooled Literature Review

**Figure 1:**
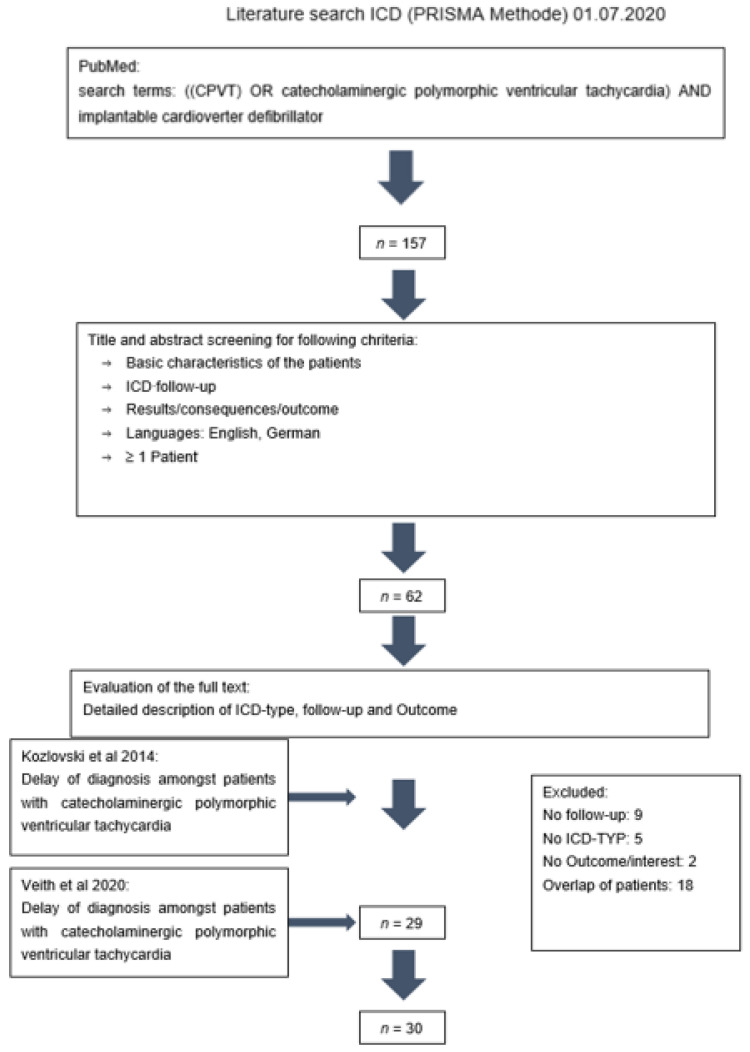
Literature review using the PRISMA methode.

To obtain the appropriate studies for our pooled analysis, we searched the PubMed, Web of Science, Cochrane Library and Cinahl database for the keywords ‘((CPVT) OR catecholaminergic polymorphic ventricular tachycardia) AND implantable cardioverter defibrillator’, Figure 1. The 157 studies resulting from this search on the first of July 2020 were screened for the inclusion criteria of having a detailed description of ICD, reported follow-up and outcome, ‘English OR German language’ and needing to consist of one or more patients. The remaining 62 articles were further reviewed regarding the same information and the ICD device type. A total of 29 studies fit our criteria [1,2,4,5,6,7,8,9,10,11,12,13,14,15,16,17,18,19,20,21,22,23,24,25,26,27,28,29,30]. Additionally, one more study was detected after reading the 28 studies and their literature. We added the patients of our previously published study to the 29 studies.

## 3. Results

### Baseline Characteristics

The 30 studies that were included in our research contained a total of 784 patients. Baseline characteristics are illustrated in Table 1. The mean age of patients was 11.3 years at admission to the hospital. The leading symptoms for hospital admission were syncope in 260 patients (33%) and aborted cardiac arrest in 113 patients (14%). Three patients (0.4%) presented atrial arrhythmias. An ICD was implanted in 337 (43%) of the 784 patients. These patients were on average 17.4 years old at date of implantation.

The cause for implantation was primary prevention in 157 patients (48%) and secondary prevention in 180 (52%). In 131 patients (39%) an appropriate ICD shock was administered in response to a VA. A total of 101 patients (30%) suffered from device related complications and 5 (1%) died from SCD despite the ICD implantation. A total of 130 patients (39%) had supportive medical treatment in addition to the ICD. A total of 125 patients (37%) were prescribed a beta blocker. Amongst these patients, 31 (9%) had a combination of a beta blocker and Flecainide. The mean follow-up time for ICD-therapy was 38.9 months.

In Table 2, a detailed overview over the number of patients with complications, which complications they had and the average follow-up time in months according to the studies are presented. Most complications that occurred were inappropriate shocks set off by different causes such as supraventricular arrhythmias or lead fracture or failure. Events of electrical storm were prominent as well.

We compared the data concerning T-ICDs and s-ICDs that were implanted in the 337 patients regarding the complications occurring in those groups (Table 3). A T-ICD was implanted in 330 patients (98%), while 7 patients (2%) had an S-ICD implanted. A total of 97 patients (29.4%) with a T-ICD experienced a total of 234 complications. Four patients (57.1%) with an S-ICD experienced a total of ten complications.

All complications that occurred in the seven patients with the S-ICD were events of inappropriate shocks, caused by T-wave oversensing in six (60%), R-wave oversensing in one (10%) and an electrode defect in one (10%) of the events. Two (20%) of the shock events happened due to an unknown cause. The S-ICD patients had no complications such as lead fracture or failure, electrical storm, thromboembolic events or endocarditis.

In comparison, 152 patients (65%) with T-ICD suffered inappropriate shock events that occurred due to unknown causes in 81 (53%) and by supraventricular arrhythmias in 65 (42.8%) cases. No events of R-wave oversensing, one event of T-wave oversensing (0.7%), four events of sinus tachycardia (2.6%) and one electrode defect (0.7%) were documented.

In comparison, we observed a significant number of lead fracture or failure (26 (11%)) and electric storm (46 (19.7%)) events for the T-ICDs. One (0.4%) thromboembolic event and two (0.8%) cases of endocarditis were also registered.

## 4. Discussion

We performed a pooled analysis which included a total of 784 patients that were diagnosed with catecholaminergic polymorphic ventricular tachycardia. We focused on the aspects of complications with different ICD implantations to compare their benefits and risks. Based on our findings, we made the following conclusions:

- ICD implantation is the leading therapy for CPVT with syncope or cardiac arrest despite getting medical treatment with beta-blockers and/or flecainide to prevent a cardiac arrest.
- The complication rate in patients with ICD is still a point to be aware of.
- While complications caused by lead failure or fracture do not occur frequently, the rate of complications connected to inappropriate shock events is very high in subcutaneous ICDs.

The S-ICD-system, introduced in 2010, allows with its extracardiac implantation a reduction of common perioperative and long-term complications that may be associated with the implantation of a T-ICD system.

As the S-ICD therapy showed a very high complication-free rate of 99% over 180 days, while in >90% of cases all episodes of ventricular fibrillation were successfully terminated [32], and had a complication free rate of 99.7% after 30 days and 98% after one year [33], it was implemented in European guidelines as an alternative to T-ICD in 2015, unless there is an overt bradycardic pacer indication [34]. Additionally, since November 2017, the American Heart Association has given a Class-I indication for S-ICD implantation in patients with difficult vascular access or a high risk of infection (and no pacemaker indication) [35].

S-ICD seems therefore according to the available data non-inferior to conventional systems regarding efficacy and safety [32,33,36].

If a young patient presents with arrhythmic events or syncope of arrhythmic origin within emotional or physical stress, CPVT should be considered and an exercise stress test should be performed. If the patient is diagnosed with CPVT, the implantation of an ICD should be considered for the patient and for other affected relatives. This is especially advised for patients who present with syncope or cardiac arrest under the first-line therapy of beta blockers. Beta-blockers are highly recommended in patients who are suffering from CPVT [2,35,37]. However, betablockers have side effects, which may lead to incompliance and/or failure of beta-blockers to stop the risk of ventricular tachyarrhythmias [24]. In addition, reducing the beta-blocker dosage due to pregnancy may reduce the successful treatment rate.

An ICD implantation in CPVT patients is recommended according to ESC guidelines in case of an aborted cardiac arrest, class I recommendation. In the present review it seems that 53% of patients who received ICD suffered initially from an aborted cardiac arrest. ICD implantation in addition to drugs is recommended in patients with a diagnosis of CPVT who experience recurrent syncope or polymorphic/bidirectional VT despite optimal therapy. The present review of papers shows that at least 56% of patients received an ICD for primary prevention related to ongoing symptoms or ventricular tachycardia at emotional or physical stress.

The first choice for an ICD implantation is often a T-ICD. S-ICD is an alternative which convinces through its easy handling for implantation, and is, due to its device type, not associated with lead-related complications. However, shock and sensing related problems occur more often.

The choice of device type should be evaluated and decided on a case-by-case basis. The S-ICD has been shown to be a valid alternative especially for patients with vascular access problems or high infection risks. If the S-ICD shows improvement in the future regarding the non-lead related complications and shock events, it has a certain advantage over T-ICD.

The role of S-ICD in patients with channelopathies, e.g., CPVT, has not been in depth investigated. In the present data the S-ICD seems to be helpful, however the inappropriate shock rate needs to be analyzed and therefore the S-ICD implantation needs to be individualized by the treating physician.

## 5. Conclusions

Lead failure or fracture and other complications associated with transvenous ICDs are a problem that cannot be denied. On the one hand, subcutaneous ICDs can present a solution to these problems. In addition, they are easier to implant and to maintain. On the other hand, they have a very high complication rate for oversensing and therefore inappropriate shock events or even insufficient shocks.

## 6. Study Limitations

Although we included studies with high numbers of patients and reached an overall large study population, the number of patients that had an S-ICD implanted was small and therefore comparison with p-values is limited. This makes part of our conclusions susceptible to random errors. In addition, we cannot exclude that bias are common relating to the heterogonous data source and pooled analysis character.

## Figures and Tables

**Table 1:** Baseline characteristics CPVT patients.

Studies (*n* = 30)	Overall (Patients *n* = 784)
ICD implantation	337
Age at implantation, mean (years)	17.4
Primary prevention	157 (47%)
Secondary prevention	180 (53%)
Single chamber	48 (14%)
Dual chamber	33 (10%)
VF zone, mean (bpm)	228
Appropriate shock, number of patients	131 (39%)
Complications	101 (30%)
Death despite ICD	5 (1%)
Medical treatment (with ICD)	130 (39%)
Beta blocker	125 (37%)
Beta blocker + Flecainide added	31 (9%)
ICD follow-up time, mean (months)	38.9

**Table 2:** Detailed description documenting ICD complications.

Number of Patients (*n* = 337)	Patients with Complications	Complication	Follow Up (Months)	Study
7	4 (57%)	Inappropriate shocks 8× Supraventricular arrhythmia 6× Electrode defect 2×	81 mean	Veith et al., 2020 [27]
1	0 (0%)	None	72	Ahmed et al., 2016 [4]
1	1 (100%)	Electrical storm 1×	21	Aksu et al., 2017 [5]
1	1 (100%)	Inappropriate shock 1× R-wave oversensing 1×	16.5	Berne et al., 2017 [6]
28	6 (21%)	Inappropriate shocks 18× Supraventricular arrhythmia 11× Lead failure 7× Electrical storm 2×	65 median	Broendberg et al., 2017 [7]
1	1 (100%)	Electrical storm 1×	30	Fagundes et al., 2010 [8]
1	0 (0%)	None	21	Griksaitis et al., 2013 [9]
3	1 (33%)	Inappropriate shocks 3×	48 mean	Haugaa et al., 2010 [10]
16	6 (38%)	Inappropriate shocks 6× Sinus tachycardia 4× Lead fracture 2×	46.8 mean	Hayashi et al., 2009 [2]
7	0 (0%)	None	32.9 mean	Hofferberth et al., 2014 [11]
1	1 (100%)	Electrical storm 8×	14	Hong et al., 2012 [12]
1	1 (100%)	Inappropriate shocks 2×	24	Jacquemart et al., 2012 [13]
1	1 (100%)	ICD-pocket infection	3.7	Kohli et al., 2019 [14]
27	4 (15%)	Inappropriate shocks 4×	57.6 mean	Kozlovski et al., 2014 [15]
1	0 (0%)	None	21	Kron et al., 2015 [16]
1	1 (100%)	Inappropriate shock 1×	12	Mantziari et al., 2013 [17]
6	5 (83%)	Inappropriate shock 2× Supraventricular arrhythmia 2× Electrical storm 6×	144 median (of 5 patients)	Marai et al., 2012 [18]
24	19 (79%)	Inappropriate shocks 65× Supraventricular arrhythmia 24× Electrical storm 4× Thromboembolic event 1× Lead fracture/failure 8×	39.6 median	Miyake et al., 2013 [19]
1	1 (100%)	Inappropriate shock 1× Supraventricular arrhythmia 1×	132	Okajima et al., 2016 [20]
1	0 (0%)	None	36	Paul et al., 2014 [21]
1	0 (0%)	None	43	Pott et al., 2011 [22]
13	11 (85%)	Inappropriate shocks 24× Supraventricular arrhythmia 16× t-wave oversensing 7× Endocarditis 2× Lead fracture 2×	48 median	Roses Noguer et al., 2014 [23]
118	28 (24%)	Inappropriate shocks 21× Electrical storm 21× Lead failure 16×	NA	Roston et al., 2015 [24]
5	1 (20%)	Electrical storm 1×	24.4 mean	Schneider et al., 2013 [25]
1	0 (0%)	None	6	Seidlmayer et al., 2018 [26]
15	5 (30%)	Inappropriate shocks 5× Supraventricular arrhythmia 5×	NA	Sy et al., 2011 [22]
12	1 (8%)	Electrical storm 1×	20 median	Van der Werf et al., 2011 [31]
40	1 (3%)	Inappropriate shock 1×	60 mean	Wanguemert et al., 2015 [28]
1	0 (0%)	None	30	Yu et al., 2016 [29]
1	1 (100%)	Electrical storm 1×	53	Yu et al., 2015 [30]

**Table 3:** Comparison of complications between transvenous ICD and subcutaneous ICD.

	Transvenous ICD	Subcutaneous ICD
Number of patients	330	7
Patients with complications	97 (29.4%)	4 (57.1%)
Number of complications	234	10
Inappropriate shocks Cause of shock, *n* (%)	152 (65%)	10 (100%)
-Supraventricular arrhythmias	65 (42.8%)	0 (0%)
-R-wave oversensing	0 (0%)	1 (10%)
-T-wave oversensing	1 (0.7%)	6 (60%)
-Sinus tachycardia	4 (3%)	0 (0%)
--Electrode defect	1 (0.7%)	1 (10%)
-Lead fracture/failure	9 (6%)	0 (0%)
Electrode defect	1 (0.4%)	0 (0%)
Lead fracture/failure	26 (11.1%)	0 (0%)
Electrical storm	46 (19.7%)	0 (0%)
Thromboembolic event	1 (0.4%)	0 (0%)
Endocarditis	2 (0.8%)	0 (0%)
ICD-pocket infection	6 (2.6%)	0 (0%)
Follow-up time	46.8 months, mean (excluding studies reporting the median follow-up)	28.5 months mean 21 months median (range 16–80 months)

## Data Availability

All data will be available by contacting the corresponding author.
